# Anatomic Distal Biceps Tendon Repair With All-Suture Cortical Buttons

**DOI:** 10.1016/j.eats.2024.103128

**Published:** 2024-07-25

**Authors:** Christen E. Chalmers, Tyler R. Mange, Dean Wang

**Affiliations:** a Department of Orthopaedic Surgery, University of California Irvine, Orange, California, U.S.A.; b Department of Biomedical Engineering, University of California Irvine, Irvine, California, U.S.A.

## Abstract

Acute, traumatic distal biceps tendon ruptures are a common injury in the middle-aged athletic male population, with direct anatomic surgical repair being the most effective technique to restore maximal strength. Multiple techniques for distal biceps tendon repair have been described, including single- or dual-incision approaches and tendon fixation with cortical buttons, interference screws, suture anchors, and transosseous sutures. In this Technical Note, we demonstrate an anatomic distal biceps tendon repair technique with a single-incision approach using 2 all-suture cortical buttons.

Distal biceps tendon ruptures occur in roughly 2.55 per 100,000 patient-years in the United States, particularly in men aged 40 to 60 years.^1^ Typically, this injury occurs when an acute eccentric force is applied during elbow flexion and supination, but it can also occur from chronic repetitive overuse, leading to progressive degeneration and failure of the tendon. Other than male sex, predisposing risk factors include middle age, elevated body mass index, smoking, and steroid use.

Prior studies have demonstrated direct tendon repair is the most effective method to restore maximum forearm supination and elbow flexion strength. Several techniques have been described and include single- versus dual-incision approaches and tendon fixation with cortical buttons, interference screws, transosseous sutures, or suture anchors.^2^, ^3^, ^4^ Recent biomechanical studies have demonstrated superior fixation strength and increased load to failure with cortical button fixation compared with other methods.^5^ The short and long heads of the distal biceps tendon have distinct insertions, with the long head inserting more proximal and posterior on the bicipital tuberosity, thus allowing for unique contributions to biceps function at various forearm positions. The short head’s distal insertion allows it to be more efficient at sustaining elbow flexion when at 90° and supination when the forearm is neutral or pronated.^6^

In this Technical Note, we present a distal biceps tendon repair technique using a single-incision approach that includes anatomic repair of both the short and long heads using all-suture cortical buttons. The included video highlights the necessary steps to perform the procedure in an efficient and reproducible manner while diminishing the risks of surgical complications (Video 1).

## Preoperative Clinical Evaluation

Patients with acute distal biceps tendon ruptures often present with pain and ecchymosis in the antecubital fossa and may report a painful “pop” during the injury. The hook test should be performed with the forearm supinated and elbow held in 90*°* of flexion with the examiner using their index finger to “hook” the lateral edge of the biceps tendon to assess for disruption of the insertion on the biceps tuberosity. A reverse Popeye sign may be visualized if the lacertus fibrosus is also torn, thereby allowing the muscle belly to retract proximally. Resisted elbow flexion is often less affected than forearm supination due to compensation by the brachialis and brachioradialis; thus, strength should always be compared with the contralateral side to assess for subtle deficiencies.

A complete evaluation includes standard radiographs, which are typically normal but should be evaluated for concomitant avulsion fractures or loose bodies. Although history and physical examination are often enough to make the diagnosis, magnetic resonance imaging is useful to confirm the diagnosis and assist in assessing the degree of tendon retraction.

## Surgical Technique

### Patient Setup

**Fig 1:**
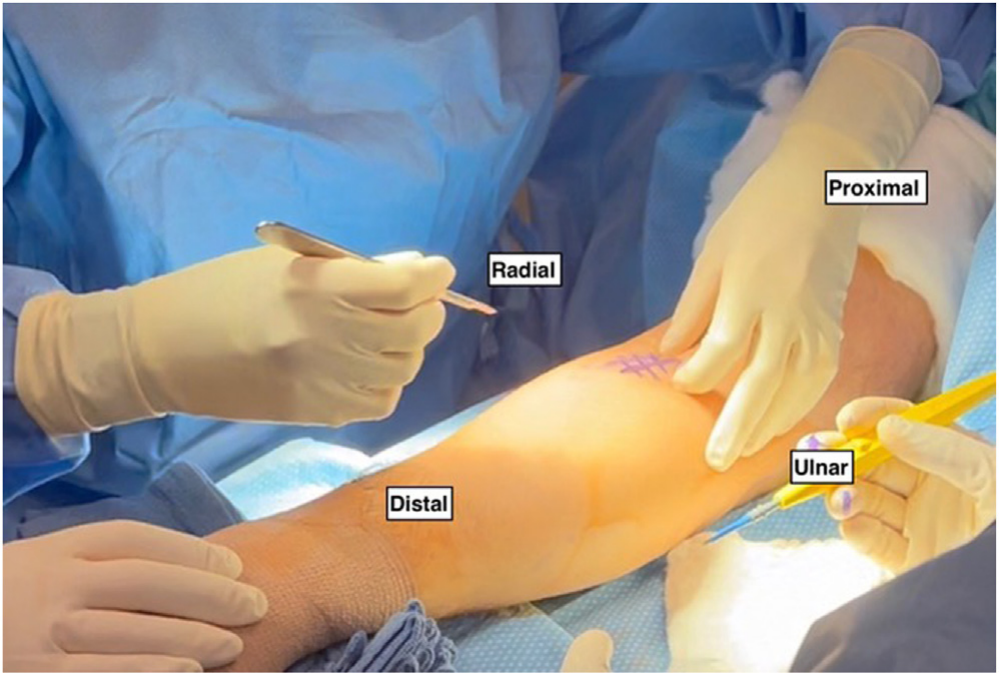
In the right upper extremity, the patient is placed supine with the arm in the center of the hand table extension. The upper extremity is prepped and draped in a standard fashion with a sterile tourniquet applied just distal to the axilla and stockinette and Coban covering the distal forearm and hand. A 3-cm volar longitudinal incision is marked out just distal to the elbow crease and headed in the direction of the bicipital tuberosity. The forearm is held in maximal supination throughout the case by an assistant to protect the posterior interosseous nerve.

In this technique, the patient is positioned supine on a standard operating room table with the arm placed in the center of a hand table extension. The upper extremity is prepped and draped in a standard fashion (Fig 1). A sterile tourniquet is applied just distal to the axilla. Throughout the case, the forearm should be maximally supinated to protect the posterior interosseous nerve.

### Initial Dissection

**Fig 2:**
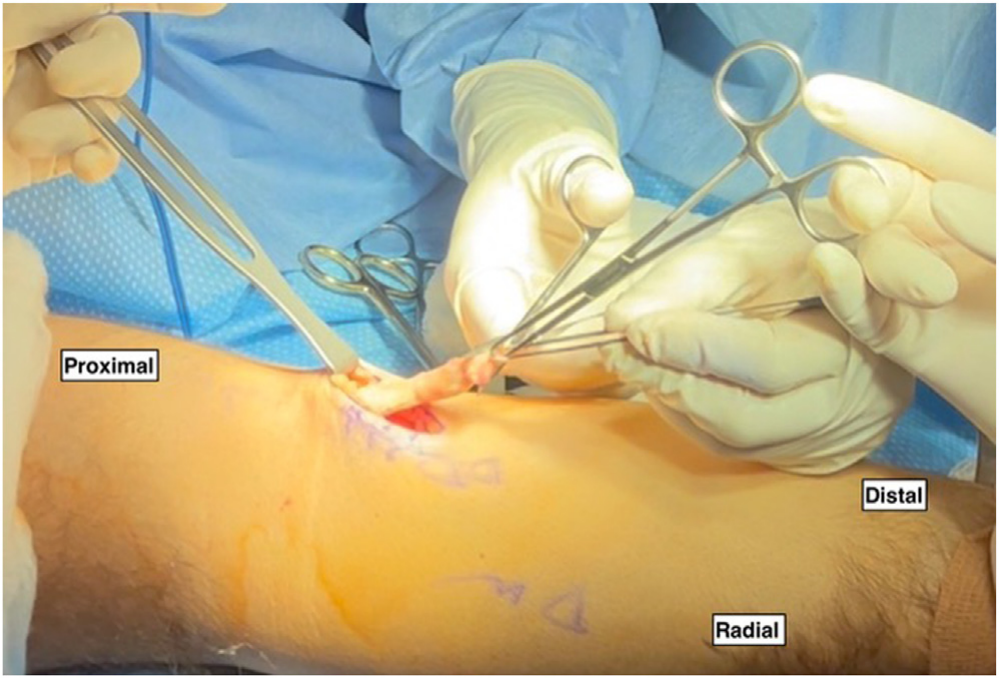
In the right upper extremity, the volar longitudinal incision is made, and the antebrachial fascia is incised. The lateral antebrachial cutaneous nerve is not always identified, yet careful retraction and dissection should be performed to protect it. Blunt dissection is used to identify the retracted biceps tendon proximally. The tendon is freed from surrounding adhesions and delivered through the incision with an Allis clamp. Using tenotomy scissors, the surrounding pseudotendon and scar tissue are debrided from the diseased distal tendon stump, and the distinct long and short heads are identified.

An approximately 3-cm volar longitudinal incision is made just distal to the elbow crease and slightly proximal but headed in the direction of the bicipital tuberosity. The lateral antebrachial cutaneous nerve is not always identified; however, careful retraction and dissection should be performed to protect it. In the acute setting, there is often a natural soft tissue dissection from the injury leading to the bare bicipital tuberosity. Using blunt dissection proximally, the retracted biceps tendon can be identified and retrieved within the superficial tissues. The tendon can be freed from any surrounding adhesions and delivered through the incision. While maintaining as much tendon length as possible, the diseased distal tendon stump is gently debrided into healthy tissue, and the distinct long and short heads are identified (Fig 2). After debridement, tendon excursion is checked to confirm that primary repair without excessive tension is possible.

### Placement of Locking Whipstitch Sutures

**Fig 3:**
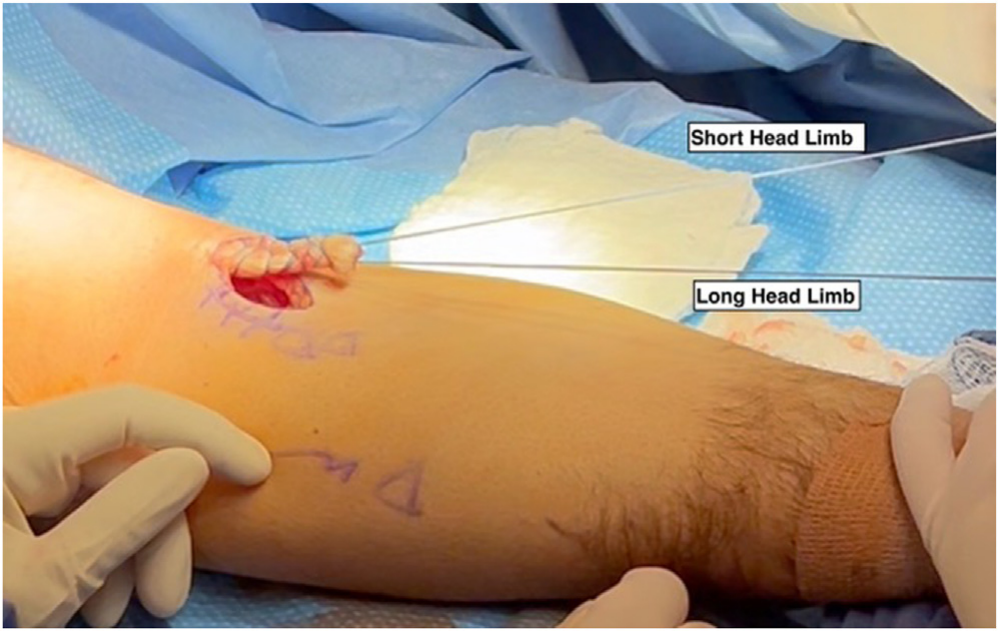
In the right upper extremity, following tendon debridement, 2 locking whipstitch loop sutures (FiberLoop; Arthrex) are placed securely through the tendon. The locking loop process is repeated 3 to 4 times each in a proximal-to-distal direction, with care taken to ensure the thick end of the suture is placed through the tendon to allow for easier limb passage through the cortical buttons. Distally, 1 whipstitch suture is exited out through the short head, while the other whipstitch suture is exited out the long head.

After tendon debridement, 2 locking whipstitch loop sutures (FiberLoop; Arthrex) are placed securely through the tendon, repeating the locking loop process 3 to 4 times each in a proximal-to-distal direction. Care is taken to ensure the thick end of the suture is placed through the tendon to allow for easier limb passage through the cortical buttons. Distally, 1 whipstitch suture is exited out through the short head, whereas the other whipstitch suture is exited out the long head (Fig 3).

### Deep Dissection

**Fig 4:**
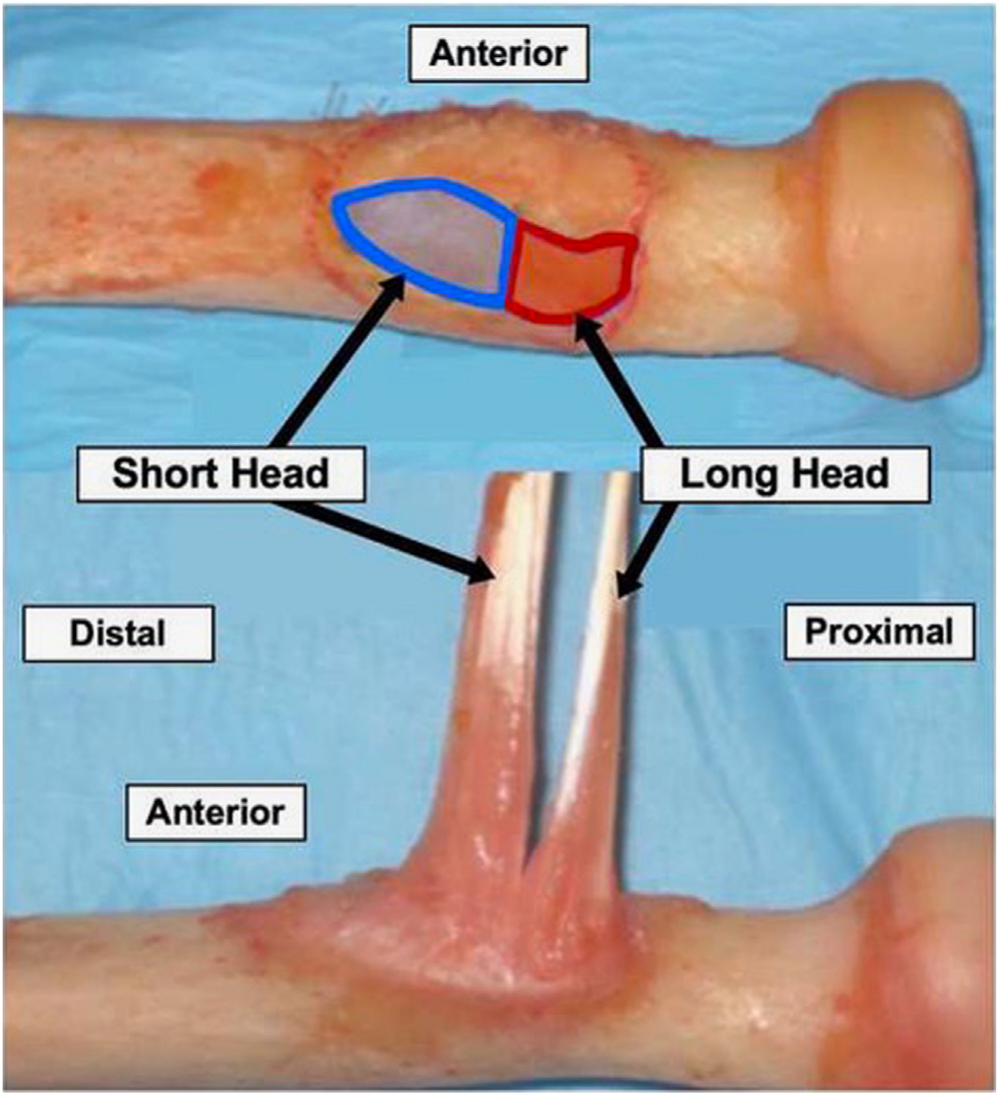
Distal biceps tendon long and short head footprints on the bicipital tuberosity in a cadaveric specimen. Following placement of the whipstitch sutures, deep dissection is continued down to the bicipital tuberosity, which is carefully exposed with retractors. The bicipital tuberosity is carefully and gently abraded of all fibrous and tendinous tissue to allow for a good healing surface and to identify the footprints of both the short and long heads.

With the arm held in maximal supination, the natural soft tissue dissection from the injury is followed down to the level of the bicipital tuberosity. The bicipital tuberosity is carefully exposed with retractors and gently abraded of all fibrous and tendinous tissue to allow for a good healing surface. Adequate exposure of the bicipital tuberosity should be achieved to allow for access to the footprints of both the short and long heads (Fig 4).

### Placement of All-Suture Cortical Buttons

**Fig 5:**
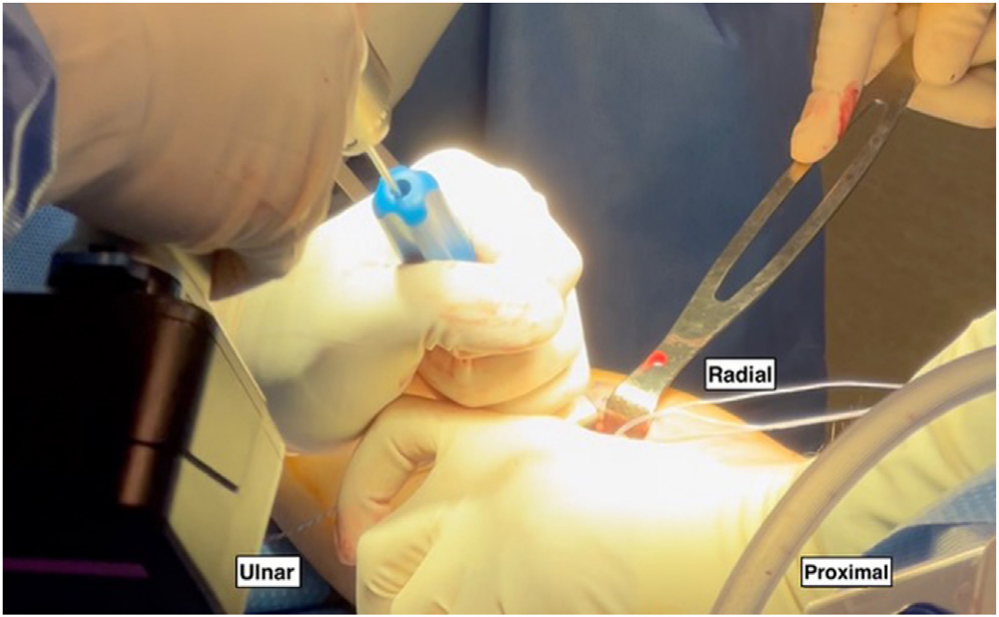
In the right upper extremity, the drill guide and a 2.6-mm drill bit for the all-suture cortical button (FiberTak button; Arthrex) is drilled unicortically on the ulnar and central portion of the short head footprint on the bicipital tuberosity. The all-suture cortical button is placed through the guide, fully malleted into the intramedullary cavity, and docked on the endosteal surface with short tugs on the suture. The linking sutures are ensured to slide easily after the button is deployed. This is repeated with another all-suture button on the ulnar and central portions of the long head footprint.

Two all-suture cortical buttons (FiberTak button; Arthrex) are placed unicortically within the bicipital tuberosity, 1 in each footprint. The drill guide is placed on the ulnar and central portions of the short head footprint on the bicipital tuberosity, and a 2.6-mm drill bit is used to open the cortex unicortically (Fig 5). The all-suture cortical button is placed through the guide and fully malleted into the intramedullary cavity. The button is then docked on the endosteal surface with short tugs on the suture. After the button is deployed, it is important to ensure the link-shuttling sutures slide easily. These steps are repeated with another all-suture button on the ulnar and central portion of the long head footprint.

### Tension Slide Technique

**Fig 6:**
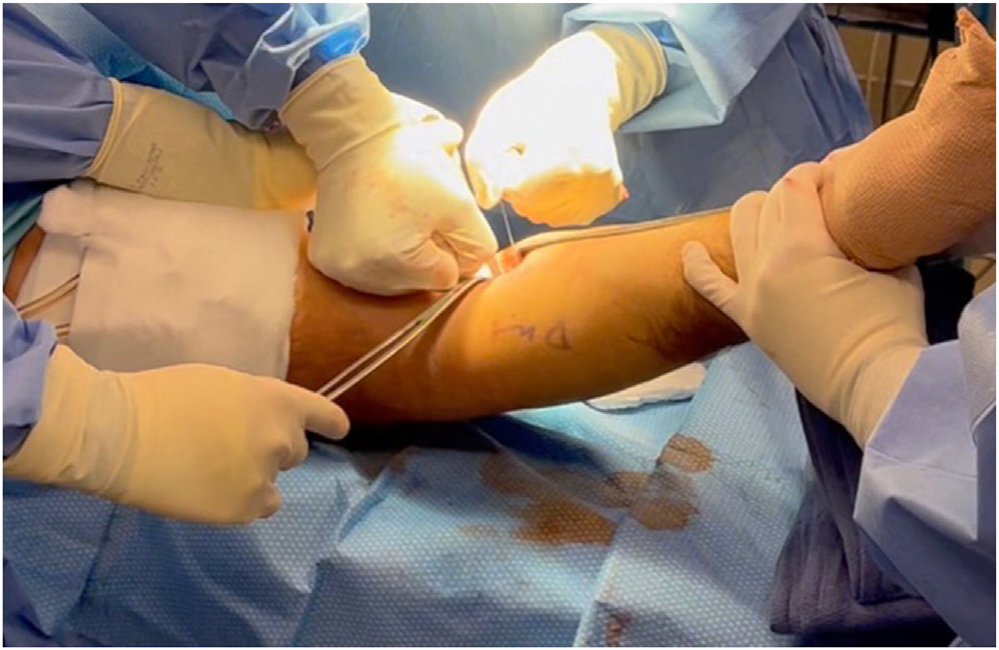
In the right upper extremity, the whipstitch suture limb from each head is passed through the corresponding cortical button using the shuttling sutures, passing only a short section of the suture limb through the link-shuttling suture to ensure easy passage of the suture limb through the cortical button. The distal biceps tendon is reduced completely down to the bicipital tuberosity with the tension slide technique, and the sutures are tied to finalize the anatomic repair with the arm held in 30° to 60° of flexion.

**Fig 7:**
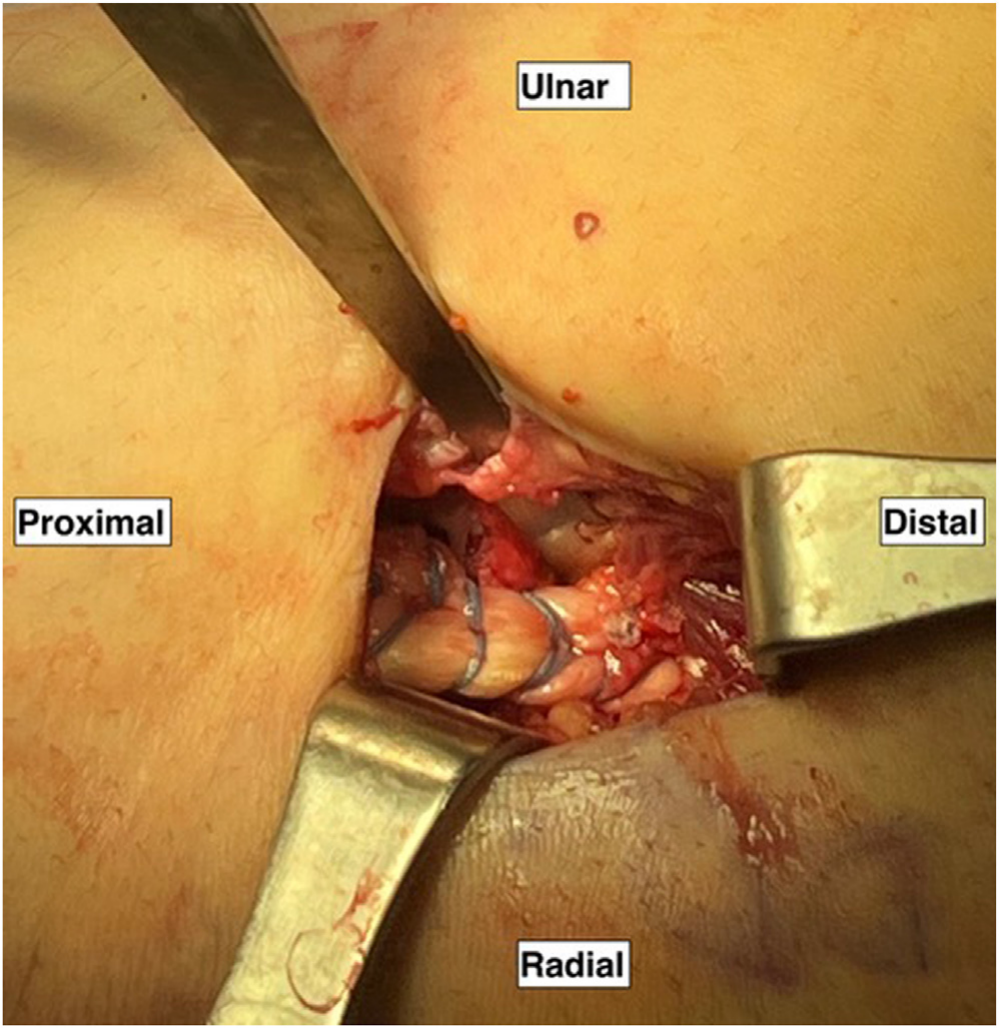
In the right upper extremity, final anatomic distal biceps tendon repair.

The suture limbs from each head are then passed through the corresponding cortical button using the shuttling sutures. Only a short section of the suture limb should be passed through the link-shuttling suture to ensure easy passage of the suture limb through the cortical button without significant friction. After all suture limbs are passed through the buttons, the distal biceps tendon is reduced completely down to the bicipital tuberosity with the arm held in 30° to 60° of flexion using the tension slide technique (Fig 6). The sutures are then tied to finalize the anatomic repair (Fig 7).

### Closure and Postoperative Care

After releasing the tourniquet and obtaining hemostasis, the repair construct is checked to ensure strong fixation and no gap formation with the arm is extended. The wound is thoroughly irrigated and closed in layers. A well-padded splint immobilizing the arm in 90° of flexion and neutral rotation is applied.

Postoperatively, a splint is maintained for 10 to 14 days and removed at the first postoperative visit. A hinged elbow brace is then applied with limited range-of-motion (ROM) parameters. Patients will remain in the hinged elbow brace until 6 to 8 weeks postoperatively, gradually increasing elbow ROM parameters to full ROM by 6 weeks postoperatively. Biceps strengthening is permitted 12 weeks postoperatively, and exercises are progressed thereafter.

## Discussion

This technique demonstrates an efficient and reproducible method for distal biceps tendon repair with a single-incision approach and anatomic repair with use of 2 small all-suture cortical buttons. This technique incorporates several advantages of previously described methods to obtain anatomic repair with 2 points of fixation within each footprint of the short and long heads while maintaining the ease of the tension slide technique. Please see Tables 1 and 2 for advantages and disadvantages, as well as the pearls and pitfalls of this technique.

**Table 1:** Pearls and Pitfalls of the Described Surgical Technique

Pearls	Pitfalls
Full supination of forearm throughout the case protects the posterior interosseous nerve while allowing for full visualization of the entire bicipital tuberosity to identify both short and long head footprints.	If pseudotendon and scar tissue are incompletely removed during the preparation of diseased distal tendon stump, the locking whipstitch sutures may not be placed in the distinct long and short heads, limiting restoration of the anatomic insertion on the bicipital tuberosity.
Check tendon excursion following tendon debridement to confirm primary repair without excessive tension is possible.	If the thick end of the whipstitch suture is not placed through the tendon, this will lead to difficulty with passing the thicker suture limbs through the cortical button.
Ensure the all-suture button shuttling sutures slide easily prior to attempting to pass the whipstitch suture limbs.	If the all-suture cortical button is not fully inserted into the intramedullary cavity, it will not properly dock on the endosteal surface, leading to button pull-out.
While using the tension slide technique to reduce the distal biceps tendon to the bicipital tuberosity and tying sutures, ensure the arm is held in 30° to 60° of elbow flexion to ensure that the tendon is reapproximated to the bone and a tension-free repair.	Crimping of a long section of the suture limb through the shuttling suture can lead to excessive friction during passage of the suture limb through the cortical button.

**Table 2:** Advantages and Disadvantages of the Described Surgical Technique

Advantages	Disadvantages
Anatomical restoration of the distinct insertions of the long and short heads of the distal biceps tendon to restore supination and flexion strength	Single incision approach poses risk of injury to the lateral antebrachial cutaneous nerve.
The smaller intramedullary all-suture cortical buttons and unicortical drilling allow for dual points of fixation to increase fixation strength while decreasing the risk of posterior interosseous nerve injury seen with bicortical implants.	Increased cost with use of 2 implants
The bicipital tuberosity prominence is maintained, which is beneficial to improve the biceps supination moment arm.	

Anatomic distal biceps tendon footprint replication may help with restoring maximal supination and elbow flexion strength. This technique anatomically restores the distinct insertions of the long and short heads of the distal biceps tendon while also increasing fixation strength through dual points of fixation. Additionally, this technique uses multiple methods to preserve the bicipital tuberosity prominence, which has been shown to improve the biceps supination moment of the arm.^7^ Prior in vivo biomechanical testing of the biceps tenodesis comparing inlay and onlay techniques demonstrated similar healing profiles, with healing occurring predominately on the cortical surface.^8^ Compared with metal cortical button devices, the all-suture cortical button is inserted through a smaller 2.6-mm drill hole, which allows for 2 points of fixation on the tuberosity without a significant iatrogenic fracture risk. The small drill holes also improve the capability to drill posteriorly at the anatomic distal biceps footprint without reducing bicipital tuberosity height, which occurs during the drilling of a large cortical hole for intramedullary tendon fixation. Finally, the all-suture button has been shown to be as strong as or stronger than other methods of fixation and allows for unicortical drilling and fixation,^9^^,^^10^ thus decreasing the risk of posterior interosseous nerve injury.

In summary, this distal bicep tendon repair method emphasizes anatomic restoration to improve postoperative function while incorporating 2 points of fixation and the ease of the tension slide technique.

## Disclosures

The authors declare the following financial interests/personal relationships which may be considered as potential competing interests: D.W. is a consultant or advisor for Newclip Technics, DePuy Synthes Mitek Sports Medicine, Vericel Corporation, and Cartilage; has received research grants from Vericel Corporation and Immunis; has received travel reimbursement from Arthrex; and has equity and stocks with Cartilage and Overture Orthopaedics. All other authors (C.E.C., T.R.M.) declare that they have no known competing financial interests or personal relationships that could have appeared to influence the work reported in this article.

## Supplementary Data

Video 1 In this Technical Note, we demonstrate a distal biceps tendon repair technique utilizing a single-incision approach with anatomic repair of both the short and long heads using two all-suture cortical buttons. This video highlights the necessary steps to perform the procedure in an efficient and reproducible manner while diminishing the risks of surgical complications. The advantages of this distal bicep tendon repair method include anatomic restoration to improve post-operative function while incorporating two points of fixation, small drill holes to limit iatrogenic fracture risk, and the ease of the tension slide technique to reduce and repair the ruptured tendon.
